# Functional connectivity changes between amygdala and prefrontal cortex after ECT are associated with improvement in distinct depressive symptoms

**DOI:** 10.1007/s00406-023-01552-7

**Published:** 2023-01-30

**Authors:** Ann-Kathrin Domke, Moritz Hempel, Corinna Hartling, Anna Stippl, Luisa Carstens, Rebecca Gruzman, Ana Lucia Herrera Melendez, Malek Bajbouj, Matti Gärtner, Simone Grimm

**Affiliations:** 1 Department of Psychiatry, Centre for Affective Neuroscience (CAN), Charité – Universitätsmedizin Berlin, corporate member of Freie Universität Berlin and Humboldt Universität zu Berlin, Campus Benjamin Franklin, Hindenburgdamm 30, 12203 Berlin, Germany; 2 Department of Psychology, MSB Medical School Berlin, Rüdesheimer Straße 50, 14197 Berlin, Germany; 3 Department of Psychiatry, Psychotherapy and Psychosomatics, Psychiatric Hospital, University of Zurich, Lenggstrasse 31, 8032 Zurich, Switzerland

**Keywords:** Resting-state fMRI, Functional connectivity, Electroconvulsive therapy, Major depressive disorder

## Abstract

Electroconvulsive therapy (ECT) is one of the most effective treatments for treatment-resistant depression. However, the underlying mechanisms of action are not yet fully understood. The investigation of depression-specific networks using resting-state fMRI and the relation to differential symptom improvement might be an innovative approach providing new insights into the underlying processes. In this naturalistic study, we investigated the relationship between changes in resting-state functional connectivity (rsFC) and symptom improvement after ECT in 21 patients with treatment-resistant depression. We investigated rsFC before and after ECT and focused our analyses on FC changes directly related to symptom reduction and on FC at baseline to identify neural targets that might predict individual clinical responses to ECT. Additional analyses were performed to identify the direct relationship between rsFC change and symptom dimensions such as sadness, negative thoughts, detachment, and neurovegetative symptoms. An increase in rsFC between the left amygdala and left dorsolateral prefrontal cortex (DLPFC) after ECT was related to overall symptom reduction (Bonferroni-corrected *p* = 0.033) as well as to a reduction in specific symptoms such as sadness (*r* = 0.524, uncorrected *p* = 0.014), negative thoughts (*r* = 0.700, Bonferroni-corrected *p* = 0.002) and detachment (*r* = 0.663, *p* = 0.004), but not in neurovegetative symptoms. Furthermore, high baseline rsFC between the left amygdala and the right frontal pole (FP) predicted treatment outcome (uncorrected *p* = 0.039). We conclude that changes in FC in regions of the limbic-prefrontal network are associated with symptom improvement, particularly in affective and cognitive dimensions. Frontal-limbic connectivity has the potential to predict symptom improvement after ECT. Further research combining functional imaging biomarkers and a symptom-based approach might be promising.

**Supplementary Information:**

The online version contains supplementary material available at 10.1007/s00406-023-01552-7.

## Introduction

Depression is the leading mental health disorder worldwide and affects 16–20% of individuals in their lifetime. It is a very heterogeneous disorder with varying symptom presentations such as low mood, anhedonia, cognitive dysfunctions, and somatic manifestations [1]. Considering the wide range of depressive symptoms, different types of antidepressant treatment (i.e., pharmacological or psychotherapeutic) may affect distinct symptom dimensions that are associated with unique neurobiological mechanisms of response [2]. Furthermore, it is possible that patients with distinct symptom profiles or depression biotypes may benefit from different antidepressant therapies [3]. For patients suffering from depression and characterized as resistant to pharmacological and psychotherapeutic treatment, ECT is another promising option for relieving depressive symptoms, with response rates of up to 50–75% [4]. Nevertheless, there are still many patients who continue to suffer from specific symptoms or do not respond to treatment with ECT at all [5]. A better understanding of the specific effect of ECT on distinct symptoms, as well as of symptom and treatment-specific biomarkers, is of paramount clinical importance, as failed treatment attempts increase patients' burden of disease and are associated with an increased risk of suicide [6–8].

We previously showed that distinct antidepressant treatments have differential effects on specific symptom dimensions, with improvements in cognitive symptoms after a single sub-anaesthetic dose of ketamine [9], whereas ECT specifically reduced affective symptoms [10]. Other studies that examined the relationship between specific symptom dimensions and overall ECT response found that core symptoms such as depressed mood and anhedonia improved more with ECT than somatic or vegetative symptoms [2]. Also, factors that comprise those symptoms [11, 12] had a high predictive value for ECT outcomes. A recent imaging study investigated the relation between three depressive symptom dimensions (somatic disturbances, core mood and anhedonia, and insomnia, measured with the 17-item Hamilton Depression Rating Scale [HDRS]) and volumetric changes in brain regions that are linked to depression and reported distinct structural imaging predictors [13].

Regarding affective dimensions, dysfunctional emotion regulation has been proposed as critical for effective core symptoms of depressive disorders [14]. Further, prefrontal-limbic connectivity, as a neural substrate, is associated with both the development of emotion regulation mechanisms [15] and their alteration in depression [16–18]. Different antidepressant treatment approaches have been demonstrated to restore disturbed prefrontal-limbic balance [19–21] and abnormal activity patterns within these regions [22]. Recent studies have already shown that functional connectivity measurements can reveal differences in emotion regulation [23, 24]. Further, resting state functional connectivity (rsFC) measurements may facilitate the identification of altered prefrontal-limbic network properties following ECT [25]. A longitudinal resting-state fMRI investigation by Perrin et al. [26] identified a considerable reduction in the average global functional connectivity in and around the left dorsolateral prefrontal cortex (DLPFC) region after ECT, which was accompanied by a significant decrease in depressive symptoms. In contrast, Abbott et al. [27] identified a significantly increased pattern of functional network connectivity between the posterior default mode network and the left DLPFC in remitted vs. non-remitted patients after ECT. However, due to the small sample sizes, the results of these studies can only be considered preliminary. Cano et al. [19] proposed that an FC decrease between amygdala and subgenual anterior cingulate cortex (sgACC) in early ECT treatment phases (after the 1st ECT session) might modulate a subsequent increase between the right amygdala and DLPFC (after the 9th ECT session), that might, in turn, be associated with a clinical response after ECT completion. They used predefined target regions so that the connections from the seed regions were limited to these targets. A whole-brain analysis, as a complementary approach, might provide further information on how rsFC of the amygdala or DLPFC changes after ECT. Furthermore, even though it might shed further light on the direct treatment effects of ECT, none of the prior studies have examined the association between rsFC changes and improvement in specific symptom dimensions.

Apart from the identification of treatment-specific biomarkers, it is of great clinical relevance to identify specific predictive markers that support decision-making for treatment with ECT. Several clinical predictors, such as older age, psychotic symptoms, or higher severity of depression at baseline for treatment response have already been outlined [28–30]. Previously, we could show that especially apparent and reported sadness and inability to feel (measured with the Montgomery-Åsberg Depression Rating Scale, MADRS [31] at baseline) have predictive value for ECT outcome [10]. When considering the prognostic value of neural markers, results become more variable across studies. To date, most studies of ECT prediction have relied on structural MRI [32–35]. The sparse literature referring to FC as a predictor has been rather inconsistent. Some of the previous studies identified the DLPFC, among other regions, as a region that has predictive value for ECT treatment success [36–38]. To our knowledge, rsFC of the amygdala, unlike DLPFC, has not yet been investigated as a possible predictor for ECT treatment outcome. However, baseline connectivity of the amygdala could also add predictive value, as alterations in that region play an important role in the development of affective symptoms [39] and connectivity changes represent a biomarker for treatment success [16].

The main objective of this study was to investigate the association between rsFC changes and changes in depression severity after ECT. For this purpose, we acquired resting state fMRI data before and after a full course of ECT treatment. We used a data-driven seed-based connectivity approach at the whole-brain level and focused our analyses on bilateral DLPFC and bilateral amygdala. Specifically, we expected that rsFC changes after ECT are directly related to the improvement of symptom severity. In addition, we explored whether baseline rsFC can predict response to ECT. As a primary outcome measure, we used the MADRS total score. Moreover, with respect to the diversity of depressive symptoms we considered a four-factor structure of the MADRS proposed by Williamson et al. [40] with the factors sadness, negative thoughts, detachment, and neurovegetative symptoms, to further elucidate the relation between neural changes and specific symptom dimensions.

## Materials and methods

### Study design

All patients underwent a baseline resting state fMRI scan and clinical assessment prior to ECT treatment (T0). The treatment implied right unilateral ECT with an ultra-brief pulse device with pulse lengths of 0.25 ms (Thymatron IV System, Somatics Inc.) according to the standard protocol at the Department of Psychiatry, Charité-Universitätsmedizin Berlin, which includes three ECT sessions per week over a period of 4 weeks. Anaesthesia included propofol (approximately 1.50 mg/kg) or etomidate (approximately 0.75 mg/kg) and succinylcholine (approximately 0.75 mg/kg) was used for muscular relaxation. To control for the adequate duration, motor and electroencephalogram seizure duration was monitored. During the first ECT treatment, seizure threshold was titrated, and voltage was only modified if patients did not respond clinically or showed insufficient seizures during the course of ECT (i.e., motor response < 20 s or electroencephalogram seizure activity < 30 s). For a more detailed description of the procedure see Brakemeier et al. [41]. Resting-state fMRI and clinical assessment were repeated after the last ECT session (T1).

### Participants and clinical assessments

Participants were 21 patients (10 female, age *M* = 44.05 years, SD =  ± 11.03, range 22–60 years) diagnosed with a current treatment-resistant depressive episode in accordance with the criteria of the Diagnostic and Statistical Manual of Mental Disorders (DSM-IV) and treated with right-unilateral ultra-brief ECT at the Department of Psychiatry, Charité-Universitätsmedizin Berlin. Patients classified as “treatment-resistant”, i.e. failed to respond to two antidepressant treatment trials of adequate dosage and sufficient length of time. Regarding antidepressant medication there were no restrictions at the time of enrolment, however, medication intake was documented. Depression severity was assessed using a German version of the MADRS (Montgomery and Åsberg, 1979) conducted by a trained professional. The MADRS consists of 10 items assessing the following depressive symptoms on a 7-point scale (with 0 = no abnormality and 6 = severe): apparent sadness, reported sadness, inner tension, reduced sleep, reduced appetite, concentration difficulties, lassitude, inability to feel, pessimistic thoughts and suicidal thoughts. We used a previously established four-Factor model of the MADRS [40, 42] to further explore the relation between distinct depression symptoms and neuronal correlates during resting state. The model contains the factors sadness, negative thoughts, detachment and neurovegetative symptoms (see Table S1 in the supplementary material for detailed information about the factors). Reduction of MADRS total score of 50% or more post-ECT was defined as a response, MADRS total score ≤ 10 as remission [43]. Statistical procedures for demographic and clinical data were conducted in IBM SPSS Statistics 28 for Windows. Statistical tests are based on a significance level of *α* = 0.05. The study was carried out in accordance with the latest version of the Declaration of Helsinki and approved by the Institutional Review Board of Charité-Universitätsmedizin Berlin. All participants provided written informed consent before participation.

### FMRI data acquisition

Functional imaging was conducted with a 3 T Tim Trio MR scanner (Siemens, Erlangen), a standard 12-channel head coil at the Center for Cognitive Neuroscience Berlin (Free University Berlin), using standard echo planar imaging sequences. Data were collected in 8-min runs (210 vol) with 37 oblique axial slices of 3 mm (TE = 30 ms; field of view = 192 mm, 3 × 3 mm in-plane resolution, TR 2300 s, flip angle 70°). A 3-dimensional T1-weighted anatomical scan was obtained for structural reference.

### Brain connectivity analyses

All resting state fMRI data were analyzed in Matlab (Version R2015b) using SPM12 and the CONN toolbox (Version 20.b; https://www.nitrc.org/projects/conn [44]). Preprocessing of functional and structural data was done with CONN’s default preprocessing pipeline to MNI-space. The pipeline includes motion correction (realignment and unwarping), slice-timing correction, structural segmentation and normalization, functional normalization, outlier detection (ART-based scrubbing), and spatial smoothing (8 mm). During the denoising step in CONN single-subject linear regression analyses were performed to remove the effects of head motion (12 total motion covariates: 6 motion parameters plus 6 temporal derivatives), physiological artifacts (10 total CompCor eigenvariates: 5 each from eroded WM and CSF masks), and artifactual scans. The resulting residual blood oxygen level-dependent (BOLD) time series were band-pass filtered (0.01–0.1 Hz). We followed a seed-based approach to assess ECT effects on regions of the emotional and cognitive control network. Seeds were selected based on recent literature [19, 36, 45, 46]. Seed regions of interest (ROI: x, y, z, in Montreal Neurological Institute [MNI] space) included bilateral DLPFC (± 40 36 32) and bilateral amygdala (± 24 − 2 − 20). Seed-based analyses were performed using spherical ROI templates with a diameter of 10 mm, that was built according to automated term-based meta-analyses on neurosynth.org. Single-subject seed-to-voxel correlation maps were calculated by extracting the residual blood oxygen level-dependent (BOLD) time course from the seed and computing Pearson’s correlation coefficients between that time course and the time course of all other voxels.

Statistical group analyses were carried out in two steps. First, we focused on the association of rsFC changes with symptom improvement at the end of the acute ECT phase. Linear regression analyses were implemented in CONN by defining a simple main effect of MADRS percent symptom reduction (defined as psr = (T0 − T1)/T0 × 100) as between-subjects contrast, and time point (pre vs. post) as between-conditions contrast. The same analyses were performed using the baseline scans only, to investigate the predictive power of baseline rsFC. Statistical thresholds were set to *p* < 0.001 (uncorrected) at the single voxel level and to *p* < 0.05 (FDR corrected) at the cluster level. The mean FC levels of each ROI were extracted with the REX Toolbox (https://www.nitrc.org/projects/rex/) [47]. To further explore the association between the improvement of specific symptoms and changes in rsFC post-hoc correlational analyses (Spearman’s correlation coefficient, two-sided) with the four MADRS factors were performed. All results are Bonferroni corrected, yet we also report exploratively the uncorrected results.

## Results

### Clinical and demographic data

**Fig. 1:**
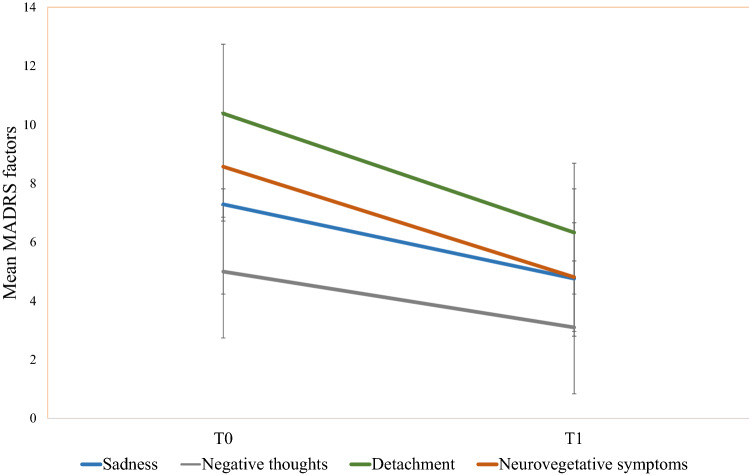
Symptom reduction in MADRS dimensions. The different lines represent the absolute change in the MADRS dimensions between (T0) and post-treatment with ECT (T1). Error bars represent standard deviations

Demographic and clinical data as well as further information regarding treatment, response, and remission are shown in Table 1. For a detailed description of diagnosis type, psychiatric and somatic comorbidities, and antidepressant medication please see tables S2 and S3 in the supplemental information. 85.71% of the patients (18/21) showed a significant reduction of depressive symptoms after the completion of their individual acute ECT phase (see Fig. 1). In total 52.38% (11/21) of the patients were classified as responders. The remission rate in our sample was 19.05% (4/21).

**Table 1:**
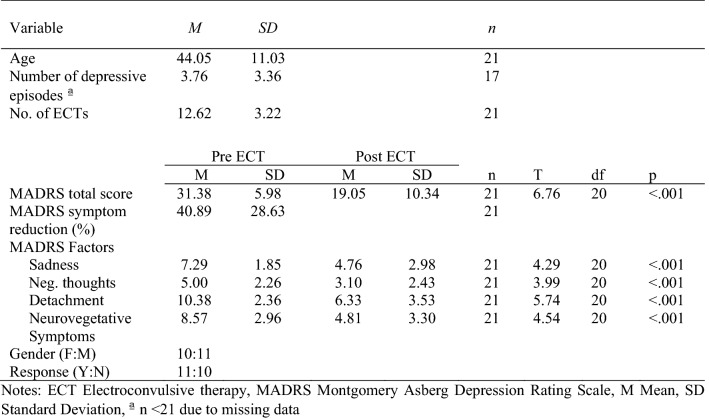
Participants demographic and clinical characteristics

PB, PB235 clone; RR, RRIM600 clone; + W, with weight application; −W, without weight application; Sulf, sulfuric acid coagulation; Form, formic acid coagulation; Nat, natural coagulation; Long dur°, maturation duration exceeding 9 days; Short dur°, maturation duration equal or shorter to 9 days

### fMRI results

**Fig. 2:**
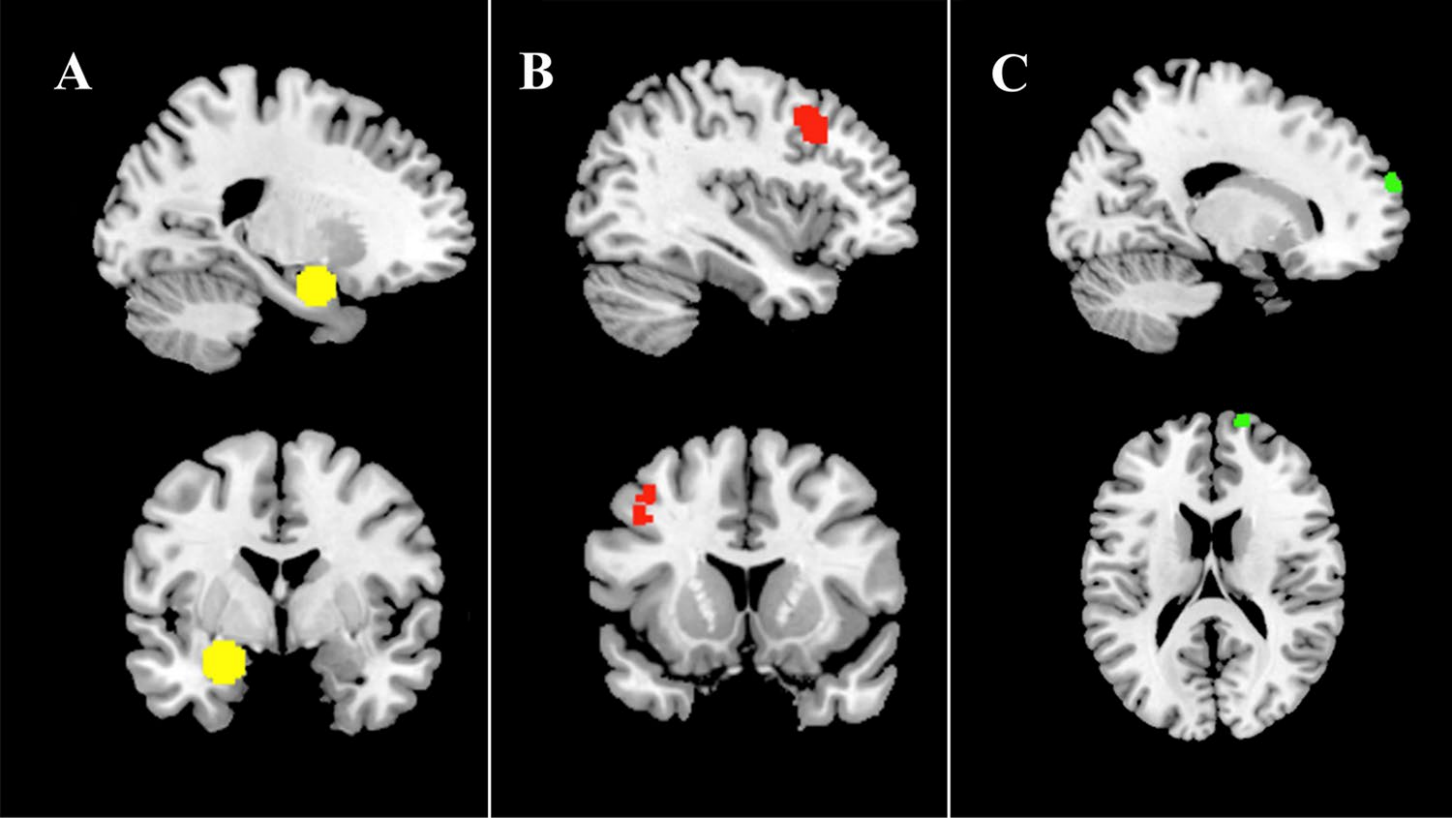
Resting-state functional connectivity (FC) related to the reduction of depressive symptoms after completion of ECT. **A** Yellow color marks the seed region in the left amygdala that was used for the seed-to-voxel analysis. **B** Red color marks the region in the left DLPFC whose FC change to the amygdala after ECT is positively related to symptom reduction (higher connectivity after ECT = higher symptom reduction). **C** Green color marks the region in the right frontal pole (FP) whose baseline FC to the left amygdala is positively related to symptom reduction (high baseline connectivity = high symptom reduction). Statistical thresholds for (**B**, **C**) were *p* < 0.001 at the voxel level, and *p* < 0.05 (FDR corrected) at the cluster level

The whole-brain regression analysis revealed a significant rsFC increase related to symptom reduction (MADRS total score) between the left amygdala seed and a cluster located in the left DLPFC (coordinates: − 38, 14, 46; Cluster size: 93; *p* = 0.008275, p-FDR corrected). To address the problem of multiple comparisons, we applied a Bonferroni-corrected alpha level of 0.0125. The result remained significant (*p* = 0.0331). See Fig. 2b for a visualization of the cluster on the cortical surface. A post hoc hierarchical multiple regression analysis in which age and gender were added blockwise as control variables revealed that both variables had no significant effect on the outcome variable MADRS total score reduction (age: *β* = 0.214, *p* = 0. 261; gender: *β* = − 0.260, p = 0. 155). The bilateral DLPFC and right amygdala seeds did not show significant rsFC changes related to symptom reduction. The analysis of baseline rsFC revealed that baseline rsFC of the left amygdala to the right FP (coordinates: 16 64 20; Cluster size: 74; *p* = 0.038816, Bonferroni-corrected *p* = 0.155264) was positively related to symptom reduction, with higher levels of baseline connectivity indicating higher levels of symptom reduction. We did not find significant effects for the DLPFC seeds.

**Fig. 3:**
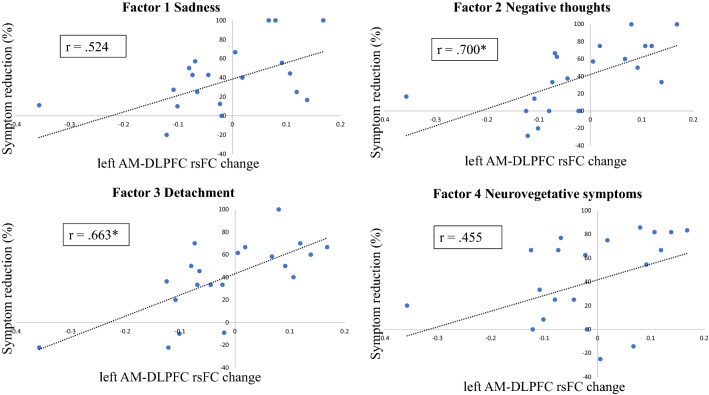
Post-hoc correlation analyses of resting-state functional connectivity (FC) change between the left amygdala and left dorsolateral prefrontal cortex (DLPFC) related to the reduction in the different symptom dimensions. Symptoms were measured with the Montgomery-Asberg Depression Rating Scale (MADRS). The *R*-value depicts Spearman’s Correlation Coefficient (* the corresponding *p*-value is < 0.05 Bonferroni-corrected)

For the significant rsFC change between amygdala and DLPFC we performed additional correlation analyses with the four MADRS factors. A strong positive relationship was observed between rsFC change (mean value of the rsFC between left amygdala and left DLPFC cluster), and the change of the factors sadness, negative thoughts, and detachment but not for the factor neurovegetative symptoms (see Fig. 3). When excluding an outlier who showed a reduction in rsFC, the positive relation between FC change and symptom reduction remained for the three mentioned symptom dimensions [sadness: *r* = 0.524, *p* = 0.059 (Bonferroni-corrected), *p* = 0.014 (uncorrected); negative thoughts: *r* = 0.700, *p* = 0.002 (Bonferroni-corrected); detachment: *r* = 0.663, *p* = 0.004 (Bonferroni-corrected)].

## Discussion and conclusion

In the present study, we investigated the modulatory effect of ECT on resting-state functional connectivity of the amygdala and the DLPFC in depressive patients. In addition to FC changes, we also investigated whether baseline FC might predict clinical outcomes. For a more detailed understanding of clinical changes after ECT, we further explored the relationship between rsFC and the specific symptom dimensions of sadness, negative thoughts, detachment, and neurovegetative symptoms. Since significant alterations in amygdala-prefrontal connectivity were reported in depression [18] and enhancement of rsFC between prefrontal-limbic regions is associated with successful treatment [48], we also expected a change in rsFC in these regions.

We found that connectivity between the left amygdala (Brodmann area [BA] 28 and left DLPFC (BA 9) increased after ECT and that these neural changes were related to overall symptom improvement. Thus, our results support previous findings from various treatment studies that found increased connectivity between prefrontal and limbic areas after successful treatment [49–51]. The theoretical framework is based on the assumption that the DLPFC, as part of the cognitive control network, modulates limbic areas such as the amygdala, a key region involved in emotion generation [52, 53]. Impairments in cognitive functioning and the negative affective bias in depression have been associated with a lack of DLPFC control [53–56]. Results of resting-state fMRI studies of amygdala alterations in depression are rather inconsistent, but reduced amygdala rsFC with the prefrontal cortex and other regions involved in emotion processing and regulation has been reported [39].

Previous studies have shown that the modulation of FC patterns involving the DLPFC is critical for the therapeutic effect of ECT [26, 27, 57]. Our key finding is in line with findings by Cano et al. [19], who observed an increase in FC between the right amygdala and right DLPFC after the ninth ECT session compared to baseline, which was also associated with symptom improvement. Based on the conceptual background, these results suggest that ECT leads to the restoration of DLPFC top-down control over the limbic system, resulting in improved emotion regulation. It is notable that we were unable to replicate the finding of decreased rsFC between the amygdala and sgACC [19]. This discrepancy, as well as the difference in laterality, could be due to differences in methodology, as Cano et al. (2016) used the DLPFC and sgACC as predefined target regions and path analysis to examine the relationship between rsFC changes and symptom improvement, whereas in this study, we used a linear regression model on the whole brain-level to avoid information loss due to predefined targets. Accordingly, we argue that treatment with ECT could impact emotion regulation directly by modulation of prefrontal-limbic FC, leading to an overall symptom improvement after the completion of ECT. Our finding further highlights the importance of prefrontal-limbic rsFC as a biomarker for ECT response. One explanation for the enhancement in rsFC could be an increase in synaptic plasticity by increasing the production of the neurotrophic growth factor BDNF (brain-derived neurotrophic factor). In a study investigating the effects of ketamine, an increase in BDNF levels after treatment was shown to be related to changes in rsFC in the prefrontal cortex, possibly reflecting synaptic plasticity effects [58]. Since several studies and meta-analyses have already demonstrated the increase in BDNF after ECT [59–61], the underlying mechanism for the increase in rsFC could be similar to that of treatment with ketamine.

To the best of our knowledge, this is the first ECT study combining resting-state fMRI with the investigation of changes in distinct depressive symptom dimensions. Depressive symptoms are quite heterogeneous, which might not only explain that about 30% of patients do not respond adequately to treatment [1, 43] but also underscores the need for a better understanding of the specific effects of a particular treatment on symptomatology. In a previous study, in which we chose a single-item rather than a factor-based approach, we observed that in particular affective symptoms, such as apparent and reported sadness and inability to feel, improved most over the course of ECT [10]. In the current sample, we observed significant improvements for all four MADRS symptom dimensions, of which changes in the dimensions of sadness, negative thoughts, and detachment were also associated with the observed change in rsFC between the amygdala and DLPFC. We did not find any correlation between the rsFC change and the change in neurovegetative symptoms. This finding further underlines that increased connectivity in the prefrontal-limbic circuit may lead to improved emotion regulation and, consequently, to a reduction in affective and cognitive symptoms. Accordingly, our results support the proposition that homogenized latent symptom dimensions from multi-item scales, as the HDRS or the MADRS, can improve the detection of imaging biomarkers that are related to the trajectories of specific symptom constellations [13].

Our baseline connectivity analyses revealed that lower rsFC between the left amygdala and right FP (BA 10) predicted higher symptom improvement at the end of treatment. Because this finding was not robust to correction for multiple comparisons, it should be considered an exploratory result. Recent studies revealed distinct connectivity patterns and various involved regions for the prediction of ECT treatment response including DLPFC [36, 38], fronto-temporal [37] and DMN [36, 62] FC, but no study so far has identified FC between FP and amygdala as a significant predictor for ECT induced symptom reduction. The underlying neurophysiological processes of FC patterns as enhancing or diminishing factors of ECT effectiveness remain elusive. It has been proposed that the electrode placement may affect the initially induced regional synchronization which leads to generalized seizure [37, 38]. It has been argued that this initiation might have a crucial impact on ECT effectiveness, as the connectivity of regions beneath the electrodes predicts ECT response [37, 38]. Our results challenge this conception since we used a temporoparietal placement with electrodes distant from the FP or amygdala. We propose that frontal and prefrontal circuits and especially their connectivity to depression-associated regions may affect ECT outcomes not solely dependent on electrode placement. Interestingly, structural [63, 64] and functional [65, 66] changes following ECT have been observed within the amygdala and within the FP [67]. On a functional level, the FP may subserve an integratory role for higher-order social, emotional, and cognitive processes [68, 69] and contribute to the development of typical symptoms associated with depression e.g. rumination [70]. Higher pre-treatment FC might have an impact on the structural and functional changes within those regions and might therefore be related to symptom reduction. The underlying effects of the initially elicited seizure quality, electrode placement and induced neuroplastic processes should be addressed in further investigations to identify ECT responders or develop individual ECT protocols.

Some limitations of this study must be acknowledged. Given the relatively small and heterogenous sample, the results of the study should be considered as preliminary. Further, clinical trials with bigger sample sizes and e.g., subgroup divisions (e.g., treatment-resistant depression (TRD) vs. non-TRD, with vs. without psychotic features, younger vs. older age) are needed to clarify direct associations between neural characteristics and response to ECT. However, with our naturalistic study design, we demonstrated that resting-state fMRI measures can provide important information about neural changes induced by ECT that are associated with symptom improvement in a sample that corresponds to clinical psychiatric reality. Furthermore, subjects received pharmacological treatment, impeding a direct interpretation of ECT effects. Nevertheless, to minimize confounding effects, medication was not modified throughout the entire ECT course. Thus, we believe that changes compared to baseline measures relate to treatment with ECT and not to pharmacological treatment. Furthermore, the simultaneous use of antidepressant and antipsychotic treatments may work in conjunction with ECT for some patients and share a similar but less effective mechanism of action [71]. Yet, future randomized controlled trials without concomitant psychopharmacological medication or the same medication for all participants in addition to ECT treatment are needed to confirm the rsFC changes shown in our naturalistic study. Our study design did not include an active control group receiving an alternative treatment (e.g., antidepressants only, ketamine, transcranial magnetic stimulation). Thus, we are unable to compare the probability of response to ECT relative to alternatives that would be relevant for making treatment decisions in clinical routine, neither can exact conclusions be derived regarding the specificity of rsFC alterations. To derive clinically relevant information from the changes in rsFC, we focused on the direct relationship between rsFC and differential symptom reduction in all our analyses. Due to the strict seed selection of ROIs with only bilateral amygdala and DLPFC, knowledge of complex network interactions is limited for this study. The aim was to highlight changes in rsFC after ECT in regions that are known to be altered in depression and that presumably play a key role in the development of cognitive and affective symptoms [72, 73].

In summary, our findings suggest that one possible mechanism of action underlying ECT may be an increased connectivity of amygdala and prefrontal cortex that is linked to the improvement of cognitive and affective symptoms in patients diagnosed with depression. We found an association between baseline amygdala and FP rsFC and symptom improvement after completion of ECT, which leads to the assumption that frontal-limbic rsFC may also be a valuable response predictor. Taken together, we propose that resting-state fMRI can be a valuable instrument in clinical routine to identify neural biomarkers such as functional connectivity in specific seed regions, that are known to be linked with specific depression symptoms such as sadness, negative thoughts, or detachment. We demonstrated that imaging biomarkers for depressive disorders can be determined in a naturalistic sample of depressed patients typically found in psychiatric units, with different primary diagnoses, comorbidities, illness durations, and from different age groups. Furthermore, we propose that a symptom-based approach, apart from categorically defined diagnoses and multi-item scale total scores, has added value for the study of a disorder as heterogeneous as depression. Further research integrating fMRI and the use of delineated symptom dimensions seems promising and may provide further insight into the underlying mechanisms of action of ECT response. Furthermore, additional scanning time points would be of great interest. Both, at an earlier time point during the acute ECT phase to investigate neuronal markers for early response, and as follow-up measurements to investigate the sustainability of ECT-induced changes in functional connectivity.

## Supplementary Information

Below is the link to the electronic supplementary material.

Supplementary file1 (DOCX 29 kb)

## Data Availability

The data that support the findings of this study are available from the corresponding author, AD, upon reasonable request.
