# Supplementary material for: Functional connectivity changes between amygdala and prefrontal cortex after ECT are associated with improvement in distinct depressive symptoms
Source: Eur Arch Psychiatry Clin Neurosci. 2023 Jan 30;273(7):1489–99. doi: 10.1007/s00406-023-01552-7 (PMC10465635; doi:10.1007/s00406-023-01552-7)
Supplement: Supplementary file 1 — Supplementary file1 (DOCX 29 kb) [file 406_2023_1552_MOESM1_ESM.docx]

Supplementary Material

**Supplementary Table S1** MADRS factor structure

| Factor | MADRS Items |
| --- | --- |
| 1 Sadness | Apparent sadness (1), reported sadness (2) |
| 2 Negative thoughts | Pessimistic thoughts (9), suicidal thoughts (10) |
| 3 Detachment | Concentration difficulties (6), Lassitude (7), Inability to feel (8) |
| 4 Neurovegetative symptoms | Tension (3), reduced sleep (4), reduced appetite (5) |

Notes: MADRS Montgomery-Åsberg Depression Rating Scale

**Supplementary Table S2** Frequencies of Clinical Characteristics

| Variable | Frequencies |
| --- | --- |
| Diagnosis Polarity | 90.5% unipolar (19)  9.5% bipolar (2) |
| Diagnosis | 4.8% F31.3: Bipolar affective disorder, current episode mild or moderate depression (1)  4.8% F31.4: Bipolar affective disorder, current episode severe depression without psychotic symptoms (1)  9.5% F32.1: Moderate depressive episode (2)  4.8% F32.2 Severe depressive episode without psychotic symptoms (1)  4.8% F33.1: Recurrent depressive disorder, current episode moderate (1)  57.1% F33.2: Recurrent depressive disorder, current episode severe without psychotic symptoms (12)  9.5% F33.3: Recurrent depressive disorder, current episode severe with psychotic symptoms (2)  4.8% F34.1: Dysthymia (1) |
| Psychiatric comorbidity | 38.1% yes (8)  61.9% no (13) |
|  | 9.5% F10-F19: past psychoactive substance use or dependence syndrome (2)  4.8% F40-F48: anxiety, stress-related, or somatoform disorders (1)  4.8% F50.-: eating disorders (1)  19.0% F60-F69: personality disorders (4) |
| Somatic disease | 52.4% yes (11) 47.6% no (10) |
|  | 14.3% E00-E89: endocrine, nutritional or metabolic (3)  28.6% G00-G99: of the nervous system (6)  4.8% H00-H95: of the eye or ear or nose (1)  4.8% I00-I99: of the circulatory system and heart (1)  4.8% N00-N99: of the genitourinary system (1) 9.5% Z00-Z99: presence of factors influencing health status (2)  33.3% G47.-: sleep disorder (7) |
| *Notes*. Census data in parentheses. Subgroups of psychiatric and somatic comorbidities refer to ICD-10 classifications. Percentage scores might not add up to exactly 100 % due to patients having more than one diagnosis. | |

**Supplementary Table S3** Antidepressant Medication Use

| Variable | Frequencies |
| --- | --- |
| Concomitant Medication | 90.5% Yes (19)  9.5% No (2) |
| Antidepressants (ADs) | 38.1% none (8)  61.9% ADs (13):  14.3% SSRIs (3)  9.5% SNRI (2)  14.3% NDRIs (3) 19.0% SARIs (4)  9.5% TCAs (2)  4.8% TeCAs (1)  4.8% MAOI (1)  4.8% NaSSA (1) |
| Other psychiatric medication | 66.7% none (14)  33.3% others (7):  28.6% antipsychotics (6)  19.0% mood stabilizer (4)  4.8% benzodiazepines (1) |

*Notes*. Census data in parentheses. SSRIs= selective Serotonin-Reuptake-Inhibitors. SNRIs= Selective norepinephrine reuptake inhibitor. NDRIs= Norepinephrine-dopamine reuptake inhibitors. SARIs= Serotonin antagonist and reuptake inhibitors. TCAs= Tricyclic antidepressants. TeCAs= Tetracyclic antidepressants. MAOIs= Monoamine oxidase inhibitors. NaSSA= Noradrenergic and Specific Serotonergic Antidepressant. Percentage scores might not add up to exactly 100 % due to patients taking more than one psychiatric medication.
